# A Cu–Pd single-atom alloy catalyst for highly efficient NO reduction†

**DOI:** 10.1039/c9sc03172c

**Published:** 2019-08-05

**Authors:** Feilong Xing, Jaewan Jeon, Takashi Toyao, Ken-ichi Shimizu, Shinya Furukawa

**Affiliations:** Institute for Catalysis, Hokkaido University N-21, W-10 Sapporo 001-0021 Japan furukawa@cat.hokudai.ac.jp +81-11-706-9163; Elements Strategy Initiative for Catalysts and Batteries, Kyoto University Katsura Kyoto 615-8520 Japan

## Abstract

A series of Cu–Pd alloy nanoparticles supported on Al_2_O_3_ were prepared and tested as catalysts for deNO_*x*_ reactions. XRD, HAADF-STEM, XAFS, and FT-IR analyses revealed that a single-atom alloy structure was formed when the Cu/Pd ratio was 5, where Pd atoms were well isolated by Cu atoms. Compared with Pd/Al_2_O_3_, Cu_5_Pd/Al_2_O_3_ exhibited outstanding catalytic activity and N_2_ selectivity in the reduction of NO by CO: for the first time, the complete conversion of NO to N_2_ was achieved even at 175 °C, with long-term stability for at least 30 h. High catalytic performance was also obtained in the presence of O_2_ and C_3_H_6_ (model exhaust gas), where a 90% decrease in Pd use was achieved with minimum evolution of N_2_O. Kinetic and DFT studies demonstrated that N–O bond breaking of the (NO)_2_ dimer was the rate-determining step and was kinetically promoted by the isolated Pd.

## Abstract 2

Highly active and selective NO reduction was achieved at low temperatures using a minimum amount of noble metal Pd.

## Graphical abstract

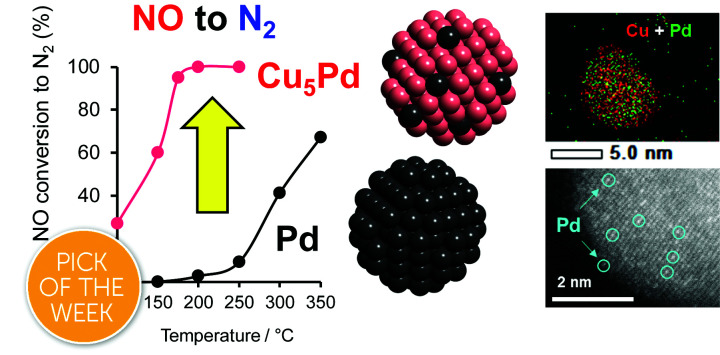

## Introduction

The reactions of nitric oxide (NO) have garnered intense interest from researchers in the human health,^1^ and bioinorganic,^2^ industrial,^3^ and environmental chemistry fields.^4^ Specifically, NO removal has long been studied as an indispensable process for exhaust-gas purification.^5^ Platinum-group metals (PGMs) such as Pt, Pd, and Rh are known to be efficient catalysts for the reduction of NO using CO,^6,7^ H_2_,^8^ NH_3_,^9^ and hydrocarbons^10^ as reductants. The recent challenges in this field involve developing catalytic systems that function (1) at low temperatures under cold-start conditions,^11^ (2) with minimum use of PGMs,^12,13^ and (3) without emitting N_2_O,^14–16^ which is a potent greenhouse gas.^17^ These issues have been individually studied using different materials. The development of a single material that enables (1)–(3) is therefore highly desirable. To the best of our knowledge, no such material has been reported. In particular, achieving both (1) and (3) is difficult because N_2_O reduction to N_2_ on PGMs requires relatively high temperatures (>300 °C).^18^ Therefore, an appropriate catalyst design is needed to obtain not only outstanding catalytic activity toward NO reduction but also high selectivity to N_2_ with minimal incorporation of PGMs.

A promising approach that overcomes these challenges is the single-atom alloying concept,^19^ which is relevant to single-atom chemistry.^20,21^ The dilution of PGM atoms with less active metal atoms not only substantially reduces the use of PGMs but also enables drastic modification of the electronic and geometric structures for enhanced catalysis.^22^ For example, the isolation of Pt or Pd with group 11 metals (Au, Ag, and Cu) enables molecular transformations that hardly proceed in the absence of single-atom alloying, such as selective hydrogenation,^23–27^ formic acid dehydrogenation,^28^ and hydrosilylation.^29^ In these systems, the group 11 metals act as inert elements but modify the electronic and geometric factors of the PGM and, thus, its catalytic properties. Conversely, for NO reduction, the group 11 elements are known to be capable of NO activation.^30–32^ Therefore, applying the single-atom alloying concept to NO reduction systems should provide an unprecedented synergistic effect for efficient NO conversion.

In this study, we focused on Cu as a main component because of its intrinsic activity toward NO reduction and its high earth abundance. We found that Cu–Pd/Al_2_O_3_ (Cu/Pd = 5) acts as a highly efficient catalyst for NO reduction at low temperatures (>150 °C), without generating N_2_O emissions. Herein, we report both an innovative catalytic system for efficient NO reduction and novel catalytic chemistry of single-atom alloys.

## Experimental details

### Catalyst preparation

Boehmite (γ-AlOOH) was supplied by SASOL Chemicals. γ-Al_2_O_3_ was prepared by the calcination of boehmite at 900 °C for 3 h. Pd/Al_2_O_3_ (Pd: 2 wt%) and Cu–Pd/Al_2_O_3_ (Cu: 6 wt%, Cu/Pd = 1) were prepared by a conventional impregnation method. The γ-Al_2_O_3_ support was added to a vigorously stirred aqueous solution containing Pd(NH_3_)_2_(NO_2_)_2_ (Kojima Chemicals, 4.765 wt% in HNO_3_) and/or Cu(NO_3_)_2_·3H_2_O (Sigma-Aldrich, 99%), followed by stirring for 3 h at room temperature (50 ml H_2_O per gram of Al_2_O_3_). The mixture was dried under a reduced pressure at 50 °C, followed by reduction under flowing H_2_ (30 ml min^−1^) at 400 °C (Pd) or 800 °C (CuPd) for 1 h. The Cu/Al_2_O_3_ (Cu: 6 wt%) and Cu–Pd/Al_2_O_3_ (Cu: 6 wt%, Cu/Pd = 3 and 5) catalysts were prepared by a deposition–precipitation method using urea. The γ-Al_2_O_3_ support was added to a vigorously stirred aqueous solution of Cu(NO_3_)_2_·3H_2_O (50 ml H_2_O per gram of Al_2_O_3_). Then, an aqueous solution of urea (Kanto, 99%) was added dropwise to the stirred mixture at room temperature (urea/Cu = 30). The mixture was sealed with a plastic film and kept with stirring at 70 °C for 10 h. After completing the precipitation of Cu(OH)_2_, the supernatant was decanted and the resulting crude product was washed with deionized water three times, followed by drying under a reduced pressure at 50 °C and calcination at 500 °C for 1 h. For Cu/Al_2_O_3_, the resulting CuO/Al_2_O_3_ was reduced under flowing H_2_ at 400 °C for 1 h. For Cu–Pd/Al_2_O_3_ (Cu/Pd = 3 and 5), the resulting CuO/Al_2_O_3_ was used for successive impregnation of Pd in a similar fashion to that mentioned above. The resulting Pd–CuO/Al_2_O_3_ was reduced under flowing H_2_ at 400 °C for 1 h.

### Reaction conditions

The catalyst (0.05 g) diluted with quartz sand (1.95 g, Miyazaki Chemical, 99.9%) was treated under flowing hydrogen (50 ml min^−1^) at 400 °C for 0.5 h prior to the catalytic reactions. NO reduction by CO was performed in a fixed-bed continuous flow system by feeding NO (5000 ppm), CO (5000 ppm), and He (balance) with a total flow rate of 96 ml min^−1^ (GHSV = 80 000 h^−1^). The gas phase was analyzed using an online thermal conductivity detection gas chromatograph (Shimadzu GC-8A, column: SHINWA SHINCARBON ST) located downstream. A stability test was done using twice the amount of catalyst (0.10 g) under similar conditions (GHSV = 40 000 h^−1^). After a time-on-stream of 24 h, the catalyst was regenerated by flowing hydrogen (50 ml min^−1^) at 400 °C for 0.5 h, followed by continuing the catalytic run. A kinetic study was performed by changing the concentration of NO and CO between 0.3 and 0.6% with that of the counterpart fixed at 0.5%. The reaction temperature was maintained at 150 °C so that NO conversion did not exceed 30%, and the reaction rate (mol s^−1^ mol_Pd_^−1^) was calculated on the basis of NO conversion. NO + CO + O_2_ and NO + CO + O_2_ + C_3_H_6_ reactions were performed under stoichiometric conditions as follows: NO (5000 ppm), CO (10 000 ppm), O_2_ (2500 ppm), He (balance) with a total flow rate of 96 ml min^−1^ (GHSV = 80 000 h^−1^), and NO (5000 ppm), CO (5000 ppm), O_2_ (5625 ppm), C_3_H_6_ (1250 ppm), and He (balance) with a total flow rate of 96 ml min^−1^ (GHSV = 80 000 h^−1^), respectively.

### Characterization

The crystal structure of the prepared catalyst was examined by powder X-ray diffraction (XRD) using a Rigaku MiniFlex II/AP diffractometer with Cu Kα radiation. High-angle annular dark field scanning transmission electron microscopy (HAADF-STEM) was carried out using a JEOL JEM-ARM200 M microscope equipped with an energy dispersive X-ray (EDX) analyzer (EX24221M1G5T). STEM analysis was performed at an accelerating voltage of 200 kV. To prepare the TEM specimen, all samples were sonicated in ethanol and then dispersed on a Mo grid supported by an ultrathin carbon film.

The Fourier-transformed infrared (FT-IR) spectra of adsorbed CO were obtained with a JASCO FTIR-4200 spectrometer equipped with an MCT detector in transmission mode (resolution 4 cm^−1^). The samples were prepared as self-supporting wafers (2.0 cm diameter, <0.5 mm thickness) and were placed inside an IR cell with CaF_2_ windows. A custom glass manifold was connected to the cell to control the gas for pretreatment and the amount of CO introduced. The cell was first purged with He, and the sample was reduced under flowing hydrogen (50 ml min^−1^) at 400 °C for 30 min. After reduction, the wafer was cooled to 40 °C under flowing He. The wafer was exposed to CO (0.5%) and He (balance) with a total flow rate of 50 ml min^−1^ for 20 min. After the CO exposure, He was flowed for 5 min to remove the gas phase and weakly adsorbed CO, followed by IR spectral measurements.

X-ray absorption fine structure (XAFS) spectra were recorded on the BL14B2 station at SPring-8 of the Japan Synchrotron Radiation Research Institute. A Si(311) double-crystal monochromator was used. Energy calibration was performed using Pd foil. The spectra were recorded at the edges of Pd K in a transmission mode at room temperature. The pelletized sample was pre-reduced with H_2_ at 400 °C for 0.5 h, and then sealed in a plastic pack under a N_2_ atmosphere together with an ISO A500-HS oxygen absorber (Fe powder). The obtained XAFS spectra were analyzed using Athena and Artemis software ver. 0.9.25 included in the Demeter package. The Fourier transformation of the *k*^3^-weighted EXAFS from *k* space to *R* space was performed over a *k* range of 3.0–15 Å^−1^. Some of the Fourier-transformed EXAFS spectra in the *R* range of 1.2–3.0 Å were inversely Fourier transformed, followed by an analysis using a usual curve fitting method in a *k* range of 3–15 Å^−1^. The back-scattering amplitude or phase shift parameters were simulated with FEFF 6L and used to perform the curve fitting procedure. For Pd–Cu scattering, intermetallic Cu_3_Pd with a *Pm*3̄*m* structure was considered for the FEFF simulation. The amplitude reduction factor (*S*_0_^2^) of Pd was determined to be 0.775 by fitting the spectra of Pd black and then used for fitting of other EXAFS spectra.

### Computational details

Periodic DFT calculations were performed using the CASTEP code^33^ with Vanderbilt-type ultrasoft pseudopotentials^34^ and the Perdew–Burke–Ernzerhof exchange–correlation functional based on the generalized gradient approximation.^35^ The plane-wave basis set was truncated at a kinetic energy of 350 eV, and a Fermi smearing of 0.1 eV was utilized. Dispersion correlations were considered using the Tkatchenko–Scheffler method with a scaling coefficient of *s*_R_ = 0.94 and a damping parameter of *d* = 20.^36^ The reciprocal space was sampled using a *k*-point mesh with a spacing of typically 0.04 Å^−1^, as generated by the Monkhorst–Pack scheme.^37^ Geometry optimization was performed on supercell structures using periodic boundary conditions. The surface was modeled based on Cu(211)-(2 × 3) (for NO and N_2_O related reactions), Cu(111)-(2 × 2) (for CO oxidation), and Cu(111)-(3 × 3) (for N_2_O decomposition) slabs that were six atomic layers thick with 13 Å of vacuum spacing. The convergence criteria for structural optimization and energy calculation were set to (a) an SCF tolerance of 1.0 × 10^−6^ eV per atom, (b) an energy tolerance of 1.0 × 10^−5^ eV per atom, (c) a maximum force tolerance of 0.05 eV Å^−1^, and (d) a maximum displacement tolerance of 1.0 × 10^−3^ Å. The transition state search was performed using the complete linear synchronous transit/quadratic synchronous transit (LST/QST) method.^38,39^ Linear synchronous transit maximization was performed, followed by energy minimization in the directions conjugate to the reaction pathway. The approximated TS was used to perform QST maximization with conjugate gradient minimization refinements. This cycle was repeated until a stationary point was found. Convergence criteria for the TS calculations were set to root-mean-square forces on an atom tolerance of 0.10 eV Å^−1^.

## Results and discussion

The monometallic Cu (6 wt%) and Pd (2 wt%) and the bimetallic Cu–Pd (Cu: 6 wt%, Cu/Pd = 1, 3, or 5; hereafter, Cu_*x*_Pd, *x* = 1, 3, or 5) catalysts were prepared using γ-Al_2_O_3_ as a support by deposition–precipitation and/or impregnation methods. X-ray diffraction (XRD) patterns of the prepared catalysts revealed that Cu–Pd solid-solution alloy phases with bimetallic compositions similar to the metal ratio in the feed were formed (Fig. S1† and Table 1).

**Table 1:** Detailed information on the catalyst prepared in this study

	Pd	CuPd	Cu_3_Pd	Cu_5_Pd	Cu
Pd loading (wt%)	2.0	10.1	3.4	2.0	0
Cu loading (wt%)	0	6.0	6.0	6.0	6.0
Cu fraction in the catalyst	0	0.50	0.75	0.83	1.0
Cu fraction in particles	0	0.44	0.72	0.89	1.0
Crystallite size/nm^a^	3.2	3.3	3.8	3.8 (4.2)^b^	4.0

a Estimated from Scherrer's equation using a Scherrer constant of 0.477 for the area-weighted mean diameter.

b Area-weighted mean diameter obtained from TEM images.

**Fig. 1:**
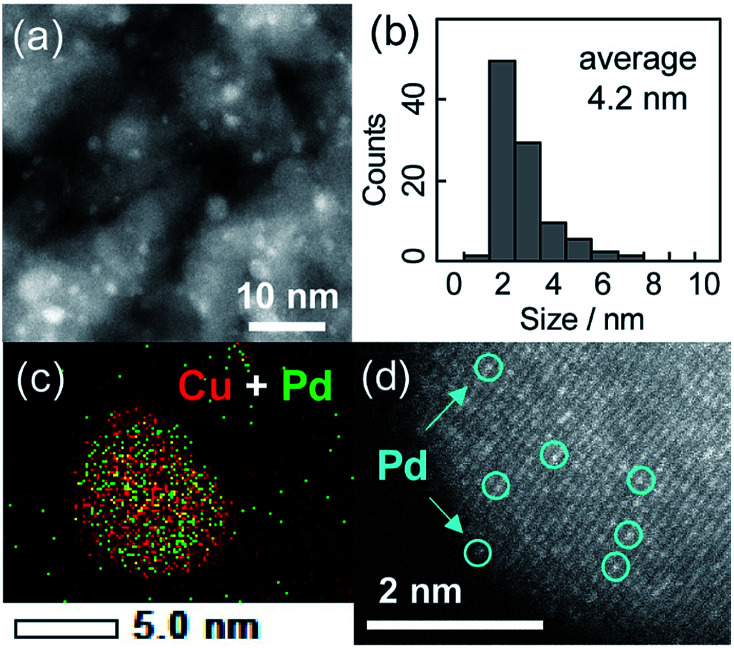
(a) HAADF-STEM image of Cu_5_Pd/Al_2_O_3_ and (b) size distribution of the nanoparticles. (c) Elemental map of the Pd + Cu overlayer, as acquired by EDS. (d) High-resolution image of a single nanoparticle.

The crystallite sizes estimated using Scherrer's equation were 3–4 nm for all of the catalysts. Fig. 1a and b show a high-angle annular dark field scanning transmission electron microscopy (HAADF-STEM) image of Cu_5_Pd/Al_2_O_3_ and the size distribution of the nanoparticles, respectively. A relatively narrow size distribution between 2 and 6 nm with an area-weighted mean diameter of 4.2 nm was obtained, consistent with the crystallite size estimated by XRD (Table 1).

The energy-dispersive X-ray spectroscopy (EDS) analysis of a single nanoparticle revealed that the Cu and Pd atoms comprising the nanoparticle were homogeneously dispersed (Fig. 1c). The high-resolution HAADF-STEM image shows an fcc crystal structure viewed along the [100] direction, consistent with the formation of a solid-solution alloy (Fig. 1d). Moreover, the isolation of Pd atoms by Cu was indicated by the presence of atoms with distinct Z contrasts. Note that the corresponding HAADF-STEM image of Cu/Al_2_O_3_ showed a weak and uniform Z contrast compared with that of Cu_5_Pd/Al_2_O_3_ (Fig. S2†).

**Fig. 2:**
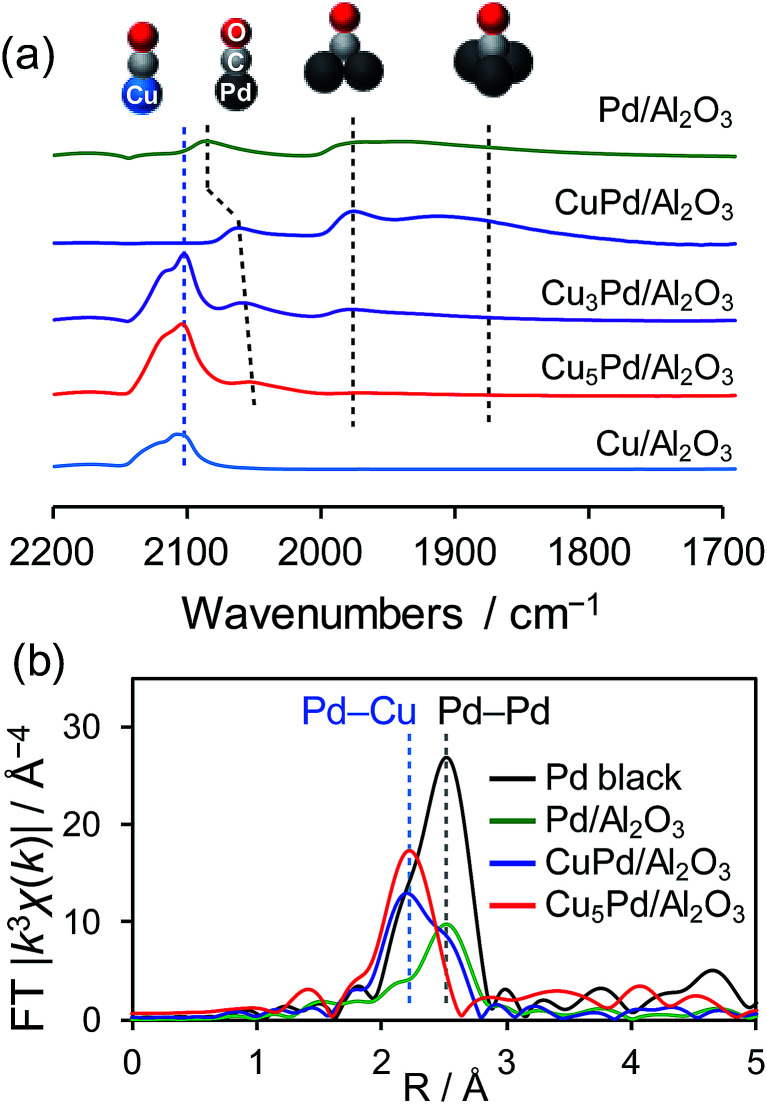
(a) FT-IR spectra of CO adsorbed on the prepared catalysts. (b) Fourier transforms of the Pd K-edge EXAFS spectra of the Pd-based catalysts.

The degree of Pd isolation was further investigated by Fourier-transform infrared (FT-IR) and extended X-ray absorption fine structure (EXAFS) analyses (Fig. 2). As shown in Fig. 2a, the FT-IR spectra of CO adsorbed onto Pd/Al_2_O_3_ exhibited absorption peaks assigned to the stretching vibration of C=O adsorbed on top (2086 cm^−1^), bridge (1975 cm^−1^), and hollow sites (∼1880 cm^−1^).^40^ Similar absorption peaks were observed for CuPd/Al_2_O_3_, suggesting that the Pd–Pd ensembles largely remain even after 1 : 1 alloying. For Cu-rich samples, an absorption band assignable to CO adsorbed on metallic Cu was also observed at 2100 nm^−1^.^41^ The peak intensities for the bridge and hollow CO substantially decreased and disappeared in the spectra of Cu_3_Pd/Al_2_O_3_ and Cu_5_Pd/Al_2_O_3_, respectively, indicating that Pd atoms at the surface were isolated upon 5 : 1 alloying.

There remained a weak absorption band for linear CO on Pd in the spectrum for Cu_5_Pd/Al_2_O_3_, suggesting that the isolated Pd atoms are also present at the surface. Fig. 2b shows the Fourier transforms of the Pd K-edge EXAFS spectra of the Pd-based catalysts (the X-ray absorption near edge structure spectra, raw EXAFS oscillations, curve-fitting, and summary of EXAFS curve fitting are shown in Fig. S3–S5 and Table S1,† respectively). CuPd/Al_2_O_3_ showed both Pd–Pd and Pd–Cu bonds, while Cu_5_Pd/Al_2_O_3_ exclusively showed Pd–Cu bonds, suggesting that the Pd atoms in the bulk were also isolated by Cu upon 5 : 1 alloying. Thus, small Cu–Pd nanoparticles with a single-atom alloy structure were successfully formed on the Al_2_O_3_ support. Considering the limited sensitivity of EXAFS and FT-IR, we cannot completely exclude the presence of Pd–Pd interaction. However, only a small number of Pd–Pd sites, if any, seem not to contribute to the overall catalytic performance.

**Fig. 3:**
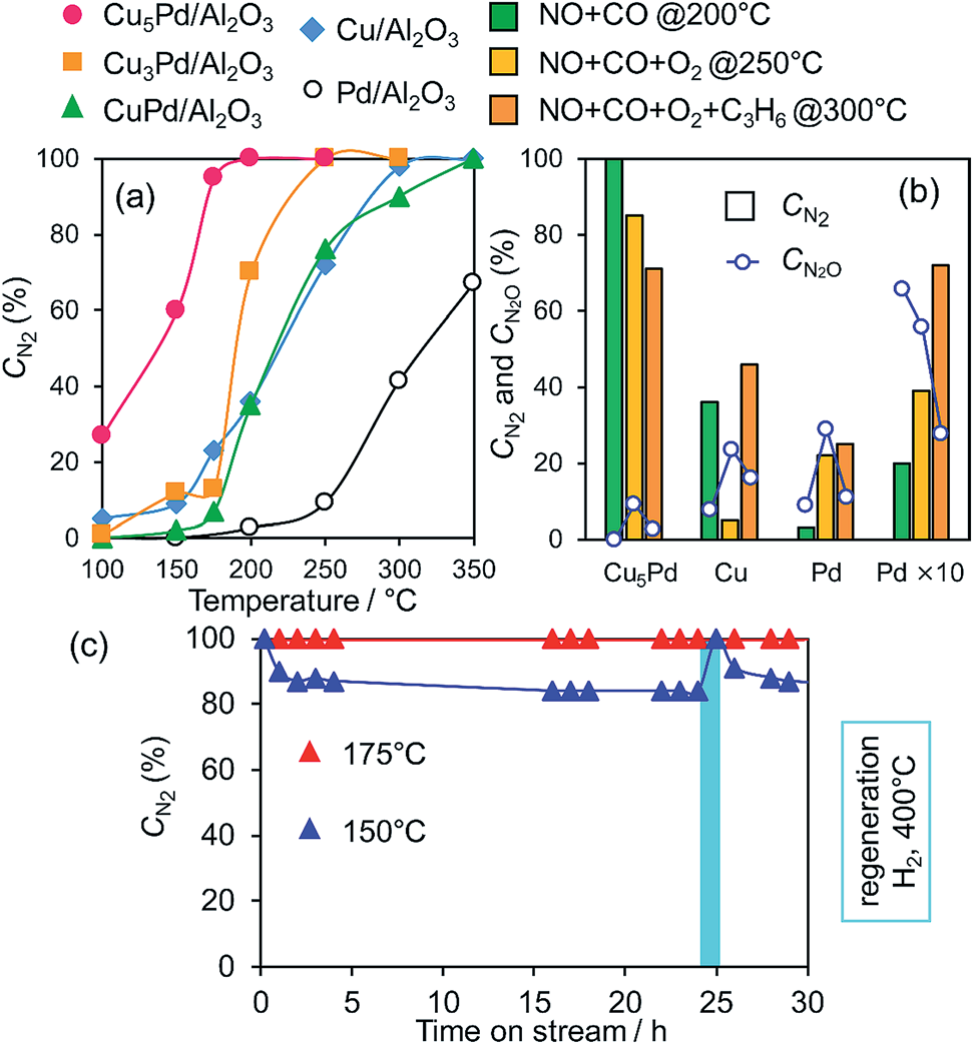
(a) NO conversion to N_2_ during the NO reduction by CO over Pd, Cu, and Cu–Pd catalysts as a function of reaction temperature (NO, CO: 0.5%, GHSV = 80 000 h^−1^). (b) Comparison between *C*_N_2__ and *C*_N_2_O_ in NO reduction in the presence of O_2_ and C_3_H_6_. (c) Stability test for Cu_5_Pd/Al_2_O_3_ in the NO + CO reaction at low temperatures (NO, CO: 0.5%, GHSV = 40 000 h^−1^).

We next tested the catalytic activity of Cu_*x*_Pd/Al_2_O_3_ in NO reduction by CO (GHSV = 80 000 h^−1^), as a model reaction for exhaust-gas purification. Fig. 3a shows the NO conversion to N_2_ (*C*_N_2__) for the prepared catalysts as a function of reaction temperature. Here, *C*_N_2__ was obtained by multiplying the NO conversion and the N_2_ selectivity (Fig. S6†). Pd/Al_2_O_3_ gave the lowest *C*_N_2__, because of the poor N_2_ selectivity <40% (Fig. S6b†).

Cu/Al_2_O_3_ exhibited a higher *C*_N_2__ than Pd/Al_2_O_3_ because of its much higher N_2_ selectivity (Fig. S6b†). CuPd/Al_2_O_3_ showed a *C*_N_2__ trend similar to that of Cu/Al_2_O_3_ because of the consequence of increased NO conversion and decreased N_2_ selectivity (Fig. S6†). Thus, the 1 : 1 alloy of Cu and Pd gave an insufficient catalytic performance for selective NO reduction. Interestingly, however, both NO conversion and N_2_ selectivity increased when the alloying ratio was increased to 3 : 1 and 5 : 1 (Fig. S6†), which resulted in great enhancements in *C*_N_2__ (Fig. 3a). NO was completely converted to N_2_ over Cu_5_Pd/Al_2_O_3_ without generating N_2_O emissions even at 200 °C, where Pd showed a *C*_N_2__ of only 5%. Notably, on going from CuPd to Cu_5_Pd, the catalytic activity increased even though the Pd content was decreased to 1/5 (Table 1). Therefore, a specific synergistic effect between Cu and Pd likely contributed to the unique properties of the single-atom alloy catalyst. We emphasize that using an excess amount of Pd/Al_2_O_3_ (0.50 g) with 10 times the equimolar Pd included in Cu_5_Pd/Al_2_O_3_ (labeled as Pd × 10) still resulted in poor performance (Fig. 3b), highlighting the outstanding performance of the single-atom alloy catalyst. We also tested the long-term stability of Cu_5_Pd/Al_2_O_3_ in NO reduction by CO under standard conditions (GHSV = 40 000 h^−1^), where 100% *C*_N_2__ was maintained at 175 °C. Although a number of bimetallic catalysts for NO reduction have been reported thus far,^12–16,42–48^ to the best of our knowledge, the present work represents the first success in complete NO_*x*_ removal at a temperature less than 200 °C. At 150 °C, although *C*_N_2__ decreased slightly at the initial stage because of N_2_O formation, it recovered after a short H_2_ treatment. This result implies that the accumulation of oxygen species at the catalyst surface triggers the loss of N_2_ selectivity and that the catalytic performance could be recovered under rich conditions. We next examined the catalytic performance of Cu_5_Pd/Al_2_O in NO reduction in the presence of O_2_ and O_2_ + C_3_H_6_; these conditions more closely resemble those encountered in practical use. Cu/Al_2_O_3_ delivered poor performance under NO + CO + O_2_ conditions. By contrast, Cu_5_Pd/Al_2_O exhibited much higher performance than Cu/Al_2_O and Pd/Al_2_O. Notably, Cu_5_Pd/Al_2_O still exhibited a performance better than or comparable to “Pd × 10” even in the presence of O_2_ or O_2_ + C_3_H_6_, respectively (Fig. 3b and S7;† a comparison with temperature dependence and *T*_50_ is presented in Fig. S7†). Furthermore, N_2_O evolution was sufficiently suppressed over Cu_5_Pd, where the *C*_N_2_O_ (NO conversion to N_2_O, Fig. 3b and S7†) was much lower than those for Pd. Thus, the single-atom alloy catalyst enabled not only a decrease in the noble metal use to 1/10 but also highly selective NO_*x*_ removal. In the reactions conducted in the presence of O_2_ and O_2_ + C_3_H_6_, reaction temperatures greater than 200 °C were needed to achieve sufficient catalytic performance (Fig. 3 and S7†). A possible explanation is that the number of active sites for NO reduction decreased because of the involvement of other reactions such as CO and/or C_3_H_6_ oxidation.

**Fig. 4:**
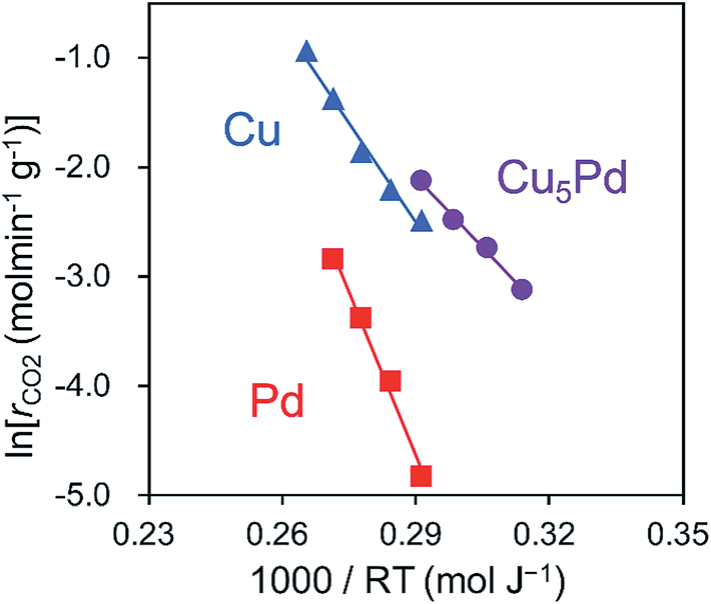
Arrhenius-type plots obtained in the NO + CO reaction over Cu_5_Pd/Al_2_O_3_, Cu/Al_2_O_3_, and Pd/Al_2_O_3_ catalysts.

**Fig. 5:**
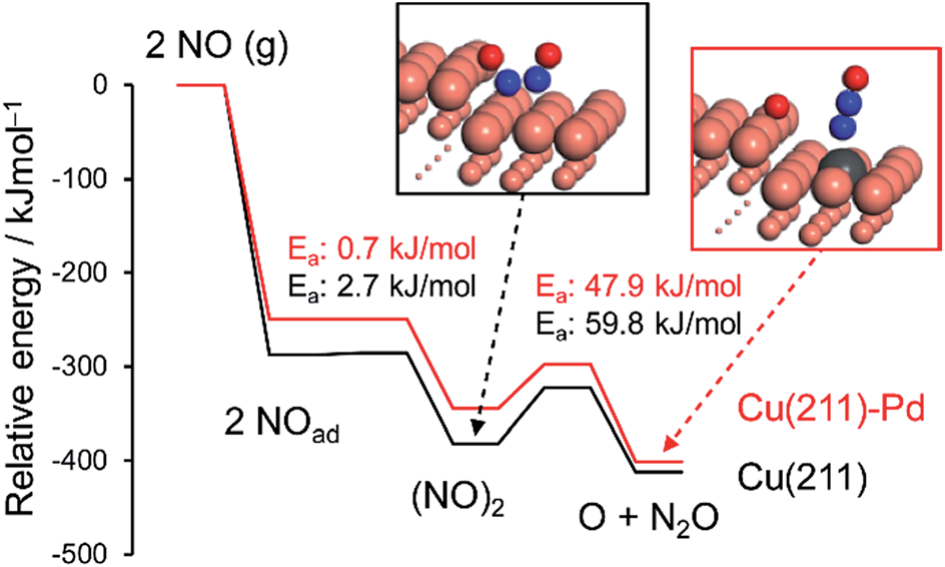
Energy diagrams of NO adsorption, dimerization, and the dimer's decomposition over pure and Pd-doped Cu(211) surfaces. The total energy of the slab and free NO was set to zero.

Next, to clarify the nature of the synergistic effect, we conducted a mechanistic study based on kinetic analysis and density functional theory (DFT) calculations. First, the apparent activation energy (*E*_A_) for NO reduction by CO was estimated for the representative catalysts. The corresponding Arrhenius-type plots and the resulting *E*_A_ values are shown in Fig. 4 and Table 2, respectively. Cu_5_Pd gave an *E*_A_ value lower than those of Pd and Cu, which is consistent with the observed catalytic activity. In addition, we estimated the reaction orders for NO and CO pressures (*P*_NO_ and *P*_CO_, respectively) to consider the rate-determining step (RDS). Both Cu and Cu_5_Pd showed negative and positive orders for *P*_NO_ and *P*_CO_, respectively (see Table 2 and Fig. S8† for details). Unlike the case for Pd-based catalysts,^49^ bond dissociation of N–O has been speculated to occur *via* (NO)_2_ dimer formation on Cu surfaces.^50–52^ Therefore, in the present study, an extended Langmuir–Hinshelwood model that includes (NO)_2_ dimer formation, the subsequent N–O scission (N_2_O formation), and N_2_O decomposition (N_2_ formation) was considered. We solved the rate equation of each step regarded as the RDS by using the overall site balance and equilibrium constants for the other steps (see the ESI,† kinetic analysis). In most cases, the reaction order for *P*_NO_ is positive, which is inconsistent with the observed experimental results. Conversely, when the N–O scission of the (NO)_2_ is considered as the RDS, the orders for *P*_NO_ and *P*_CO_ range from −2 to 0 and from 0 to 2, respectively, in agreement with the experimental values. Thus, our kinetic study suggests that the N–O scission was the RDS in NO reduction by CO. Upon incorporation of Pd atoms into pure Cu, the order for *P*_NO_ becomes less negative, while that for *P*_CO_ decreases substantially. This result indicates that the strong adsorption of NO inhibits CO adsorption onto Cu, while the latter is enhanced in the presence of Pd. We performed DFT calculations for the relevant elemental steps on pure and Pd-doped Cu surfaces. On the basis of the literature,^53^ the step site of the (211) surface was considered the active site for N–O scission (Fig. S9†). The corresponding energy diagrams are shown in Fig. 5.

**Table 2:** Activation energies estimated from experiments and from DFT calculations, along with reaction orders

	*E*_A_/kJ mol^−1^ (/eV)		
	Pd	Cu	Cu_5_Pd
Experiment	99.5	60.8	42.7
DFT: N–O	100.2^a^	59.8	47.9
DFT: CO + O	100.1^b^	60.9	34.1

Reaction order			
*P*_NO_	—	−0.27	−0.02
*P*_CO_	—	1.93	0.44

a NO dissociation at the step of Pd(511).^16^

b CO oxidation on Pd(111).^16^

NO adsorption was weakened by the addition of Pd, which is consistent with the change in the reaction orders (Table 2). Dimerization occurs at the terrace site adjacent to the step site, with a negligible energy barrier. The subsequent N–O scission is triggered by capture of an oxygen atom by the edge Cu atoms, resulting in the formation of an on-top N_2_O with *E*_A_ values of 59.8 and 47.9 kJ mol^−1^ for pure and Pd-doped Cu, respectively. The calculated *E*_A_ values agree with the experimental values (Table 2). The lower *E*_A_ for the Pd-doped Cu appears to originate from the destabilized adsorption of the (NO)_2_ dimer by Pd.

**Fig. 6:**
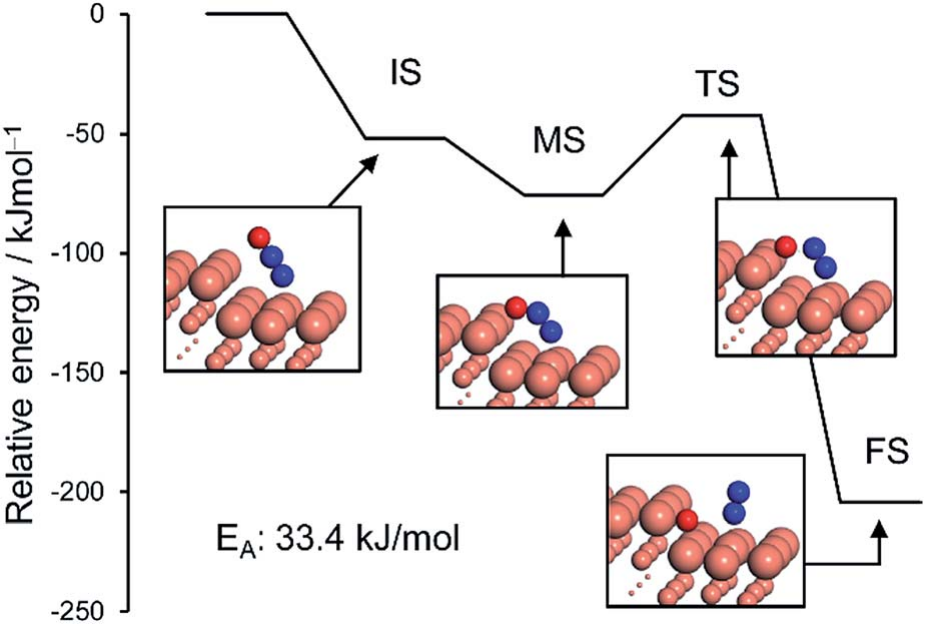
Energy diagrams of N_2_O bending (initial (IS) to intermediate (MS) states) and its subsequent decomposition to N_2_ and O (MS to final state (FS)) over the Cu(211) surface. The total energy of slab and free N_2_O was set to zero.

We also considered the CO oxidation process (CO + O → CO_2_), which is necessary for the catalytic cycle (Fig. S10†). The CO + O reaction over pure and Pd-doped Cu(111) surfaces gave *E*_A_ values of 60.9 kJ mol^−1^ and 34.1 kJ mol^−1^, respectively (Table 2 and Fig. S10†). These values are very similar to or lower than those for N–O scission, which is consistent with the RDS being the scission of N–O. We also simulated the N_2_O decomposition process (N_2_O → N_2_ + O) on Cu(211) and (111) surfaces to understand the intrinsic high N_2_ selectivity of Cu (Fig. 6 and S11; see S12† for the pictures of the optimized structures).

The monodentate linear N_2_O was bent to form a bidentate N_2_O at the edge site of the Cu(211) plane without an energy barrier. The bidentate N_2_O was subsequently decomposed into N_2_ and O with a low *E*_A_ of 33.4 kJ mol^−1^, indicating that the N_2_O once formed could be smoothly decomposed into N_2_ to afford high N_2_ selectivity. Although the Cu(111) surface was also active for N_2_O decomposition in a similar fashion, the energy barrier was higher than that of Cu(211) (51.6 kJ mol^−1^, Fig. S11†). Because large Cu–Cu ensembles are present on the surface of the Cu and Cu-rich catalysts (Cu_5_Pd and Cu_3_Pd), N_2_O decomposition could also be enhanced on these catalysts. However, for CuPd, this effect is limited because of the dilution of Cu–Cu ensembles and the increase of Pd–Pd ensembles. Thus, our calculation rationalized the substantial enhancement in catalytic activity on the basis of the formation of the Cu–Pd single-atom alloy and the origin of the excellent selectivity for N_2_ formation. The elucidated mechanism differs completely from those proposed for other bimetallic alloy systems. For example, for the Pt–Co system, Sato *et al.* reported that alloying with Co makes Pt electron-rich, which enhances back donation to adsorbed NO, inducing bond breaking.^13^ Therefore, Co likely acts as a promoter for Pt. By contrast, in our system, the isolated Pd acts as an efficient promotor for Cu.

## Conclusion

We prepared a series of Cu–Pd/Al_2_O_3_ catalysts for selective NO reduction at low temperatures. Alloying of Pd with a large amount of Cu (Cu/Pd = 5) isolates Pd and drastically improves both the catalytic activity and N_2_ selectivity, affording outstanding catalytic performance. In the NO reduction by CO, NO is completely converted to N_2_ even at 175 °C, with long-term stability for at least 30 h. The high catalytic performance is also achieved in the presence of O_2_ and C_3_H_6_, where the amount of Pd needed for a comparable performance can be reduced to 1/10, with minimum evolution of N_2_O. For Cu/Al_2_O_3_ and Cu_5_Pd/Al_2_O_3_, the N–O bond scission of the (NO)_2_ dimer is the RDS in NO reduction by CO. This step is kinetically facilitated by the isolated Pd atoms. N_2_O decomposition to N_2_ smoothly proceeds on the Cu surface, which contributes to the excellent N_2_ selectivity observed for Cu and Cu-rich catalysts. The key to this efficient catalysis is the sufficient isolation of Pd atoms by Cu, highlighting the importance of catalyst design based on single-atom alloy structures. The insights gained in this study provide not only a highly efficient deNO_*x*_ system with substantially reduced noble-metal content, but also open a new path for the chemistry of single-atom alloys.

## Conflicts of interest

There are no conflicts to declare.

## Supplementary Material

SC-010-C9SC03172C-s001
