# Supplementary material for: A Cu–Pd single-atom alloy catalyst for highly efficient NO reduction
Source: Chem Sci. 2019 Aug 5;10(36):8292–8. doi: 10.1039/c9sc03172c (PMC7006621; doi:10.1039/c9sc03172c)
Supplement: SC-010-C9SC03172C-s001 [file SC-010-C9SC03172C-s001.pdf]

Electronic Supplementary Material (ESI) for Chemical Science.
This journal is © The Royal Society of Chemistry 2019

Supporting Information

Cu–Pd Single-Atom Alloy Catalyst for Efficient NO reduction

Feilong Xing,† Jaewan Jeon,† Takashi Toyao,†,‡

Ken-ichi Shimizu,†,‡ Shinya Furukawa,*,†,‡

†*Institute for Catalysis, Hokkaido University, N-21, W-10, Sapporo 001-0021, Japan*

‡*Elements Strategy Initiative for Catalysts and Batteries,*

Kyoto University, Katsura, Kyoto 615-8520, Japan

E-mail: furukawa@cat.hokudai.ac.jp,

Tel: +81-11-706-9162, Fax: +81-11-706-9163

Table S1. Summary of EXAFS curve fitting for Pd-based catalysts.

sample	shell	N	R(Å)	ΔE (eV)	σ^2(Å^2)	R-factor
Pd black	Pd–Pd	12 (fix)	2.74 ± 0.00	6.1 ± 0.3	0.006	0.002
Pd/Al_2O_3	Pd–Pd	8.1 ± 0.7	2.73 ± 0.00	4.7 ± 0.6	0.009	0.010
CuPd/Al_2O_3	Pd–Cu	4.6 ± 0.6	2.60 ± 0.02	4.7 ± 2.5	0.004	0.004
	Pd–Pd	6.0 ± 0.5	2.65 ± 0.01	3.9 ± 0.8	0.009	
Cu_5Pd/Al_2O_3	Pd–Cu	8.4 ± 0.9	2.57 ± 0.01	2.1 ± 1.3	0.007	0.010

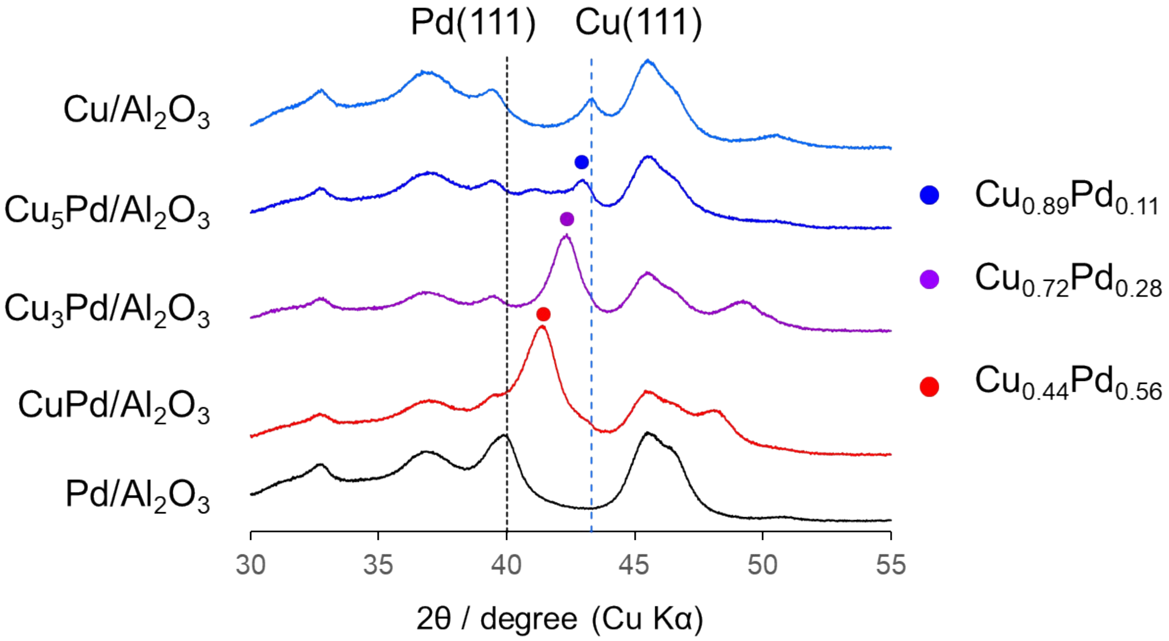

Figure S1. XRD patterns of Cu/Al_2O_3, Pd/Al_2O_3, and Cu–Pd/Al_2O_3. The Cu–Pd solid-solution alloy phases with bimetallic composition close to that of the fed ratio were formed: Cu/Pd = 1, $Cu_{0.5}Pd_{0.5}$; Cu/Pd = 3, $Cu_{0.75}Pd_{0.25}$; Cu/Pd = 5, $Cu_{0.83}Pd_{0.17}$. The diffraction peak intensity changes depending on the metal loadings as summarized Table 1.

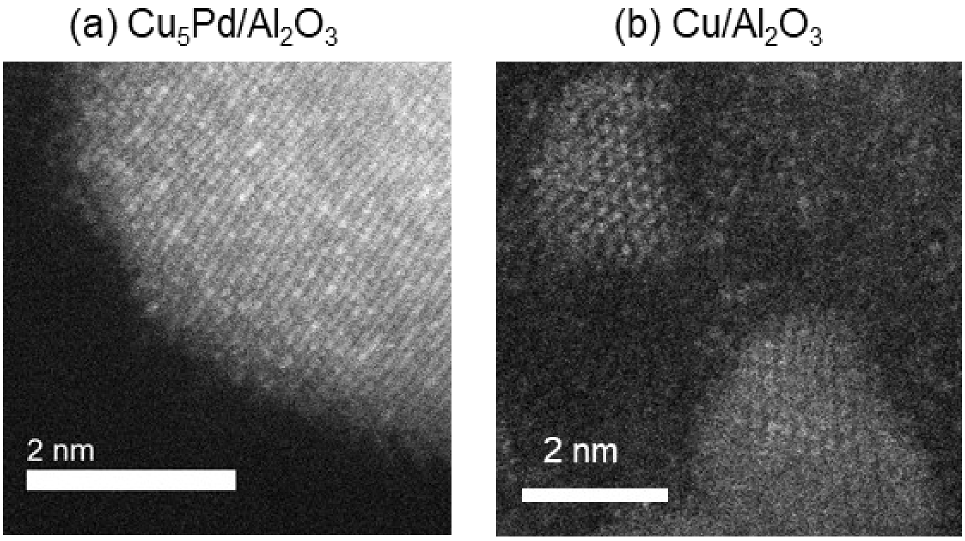

Figure S2. HAADF-STEM images of (a) Cu_5Pd/Al_2O_3 and (b) Cu/Al_2O_3.

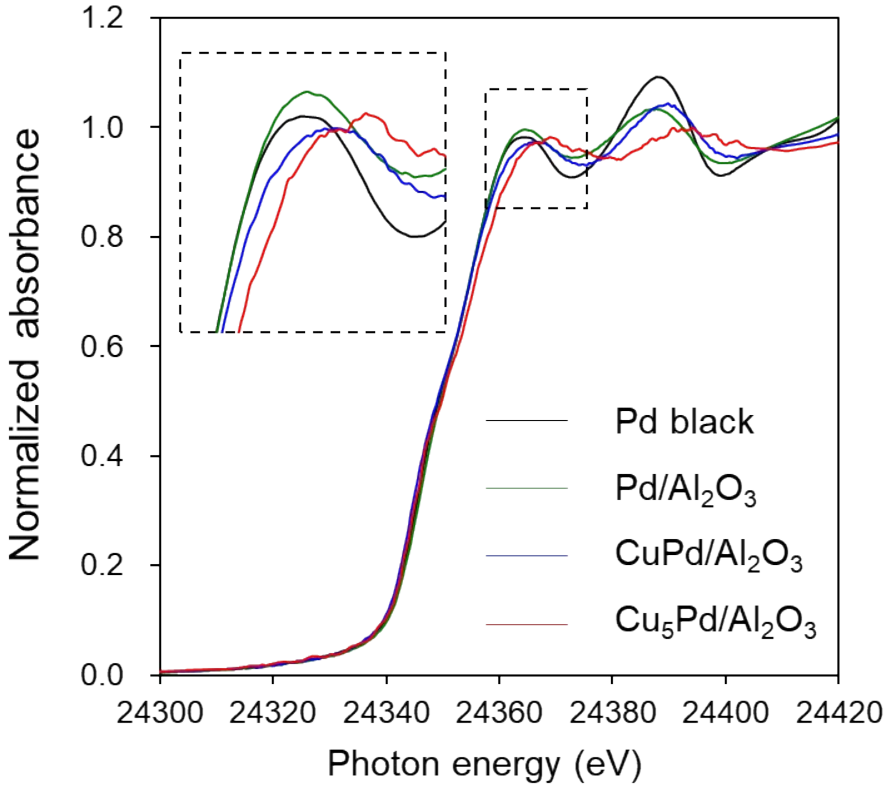

Figure S3. Pd K-edge XANES spectra of Pd-based catalysts.

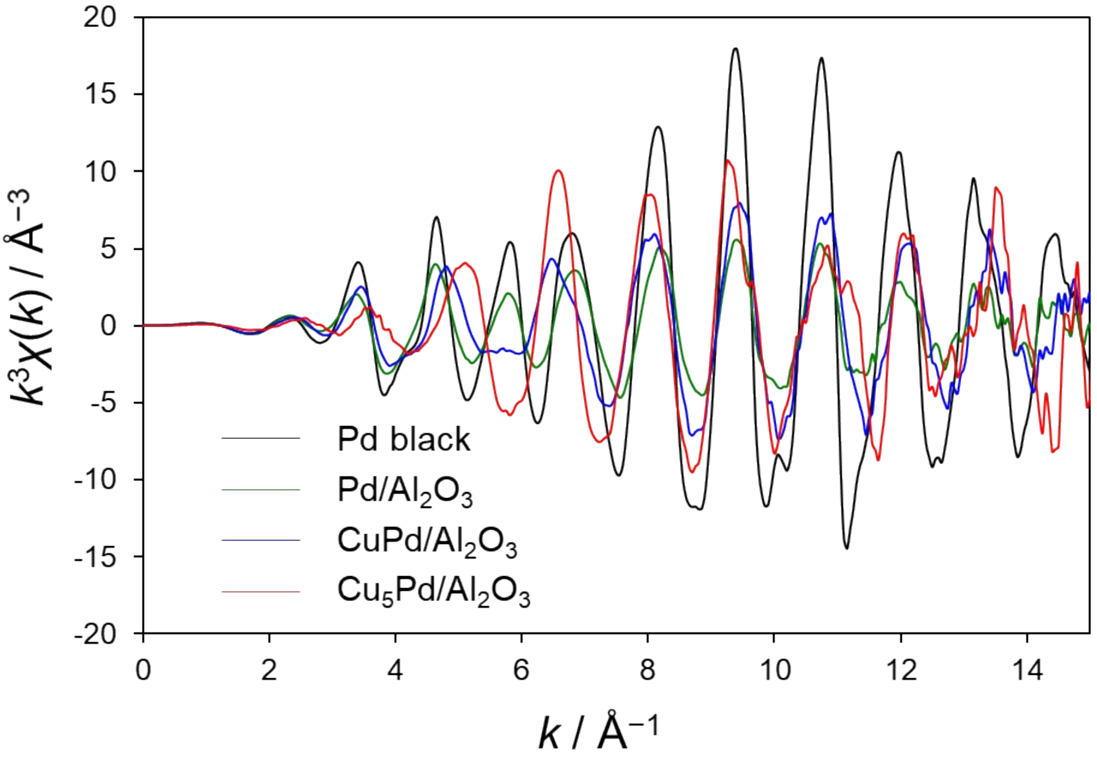

Figure S4. Pd K-edge k^3-weighted EXAFS oscillations of Pd-based catalysts.

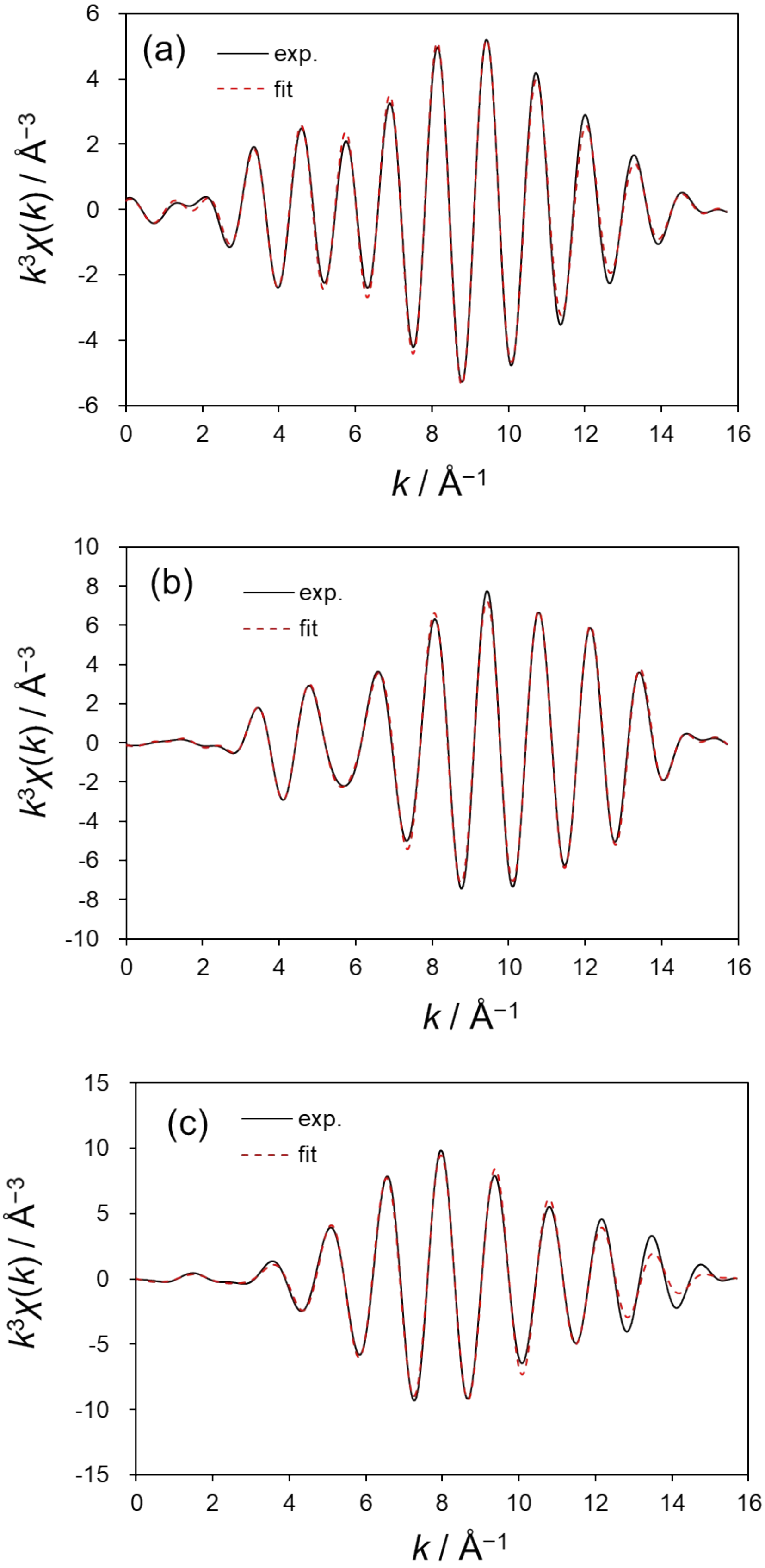

Figure S5. Fourier-filtered EXAFS oscilations (solid curve) and the curve fit (dashed line) for (a) Pd/Al_2O_3, (b) $CuPd/Al_2O_3E$,and (c) Cu_5Pd/Al_2O_3.

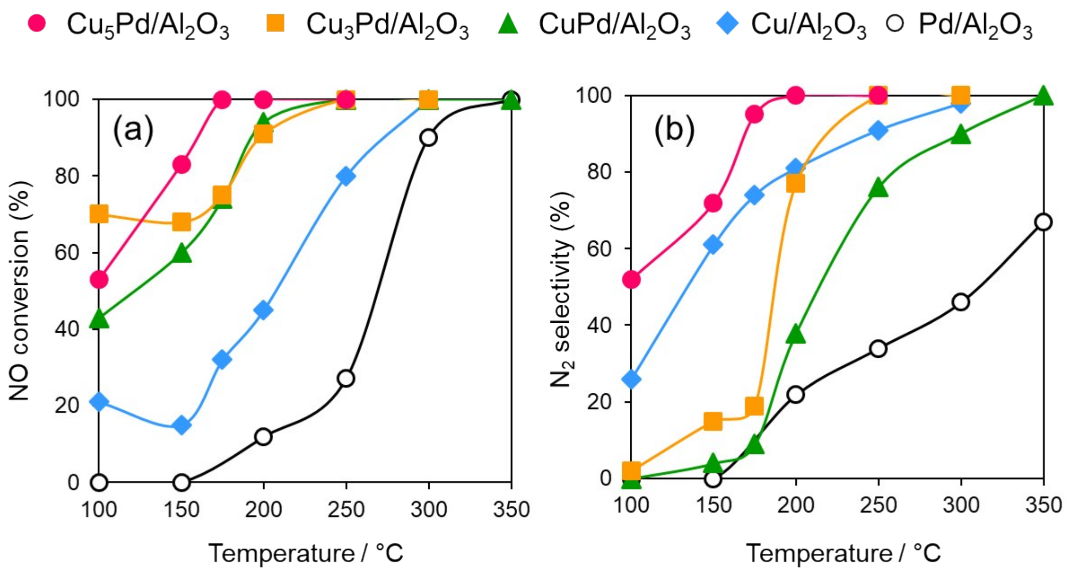

Figure S6. (a) NO conversion and (b) N_2 selectivity in NO+CO reaction over Pd/Al_2O_3, Cu/Al_2O_3, and Cu_xPd/Al_2O_3 catalysts.

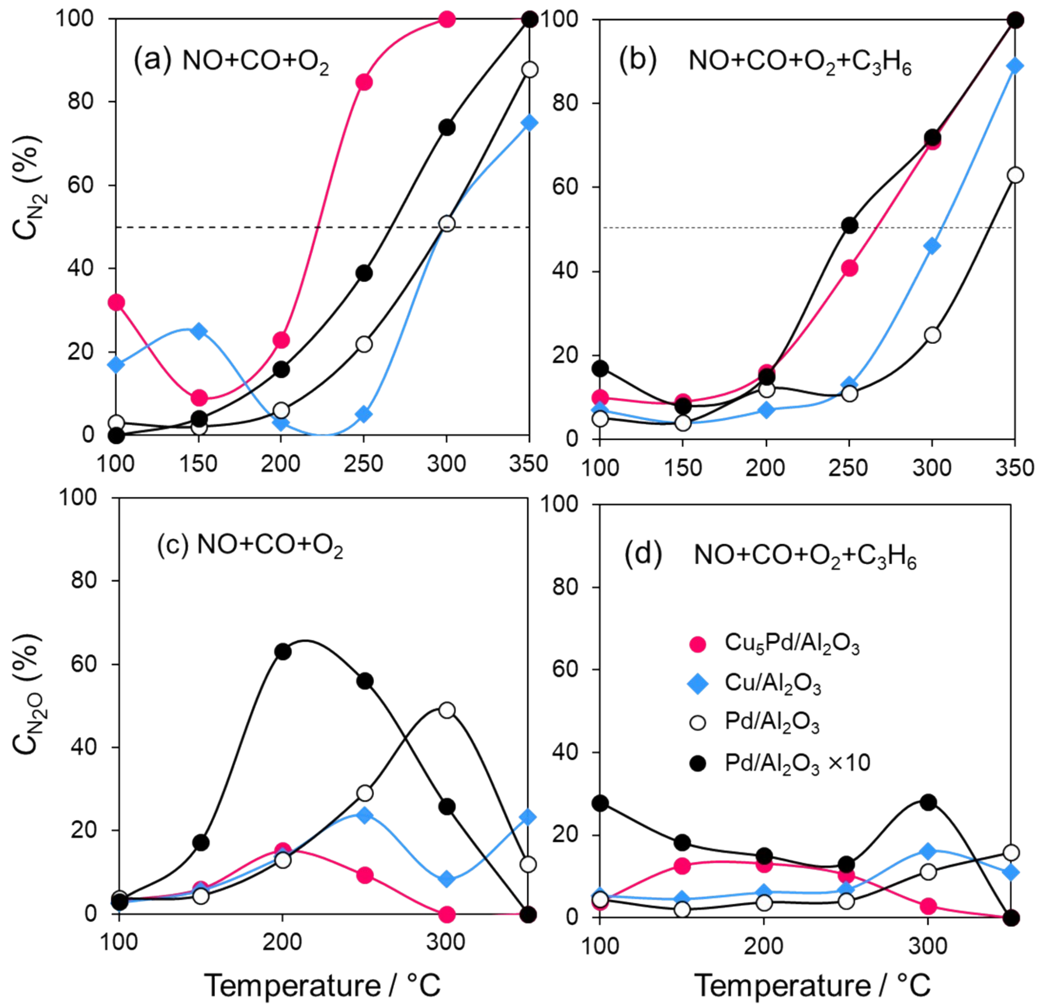

Figure S7. C_{N_2} and C_{N_2O} in $NO+CO+O_2$ and $NO+CO+O_2+C_3H_6$ reactions over Cu_5Pd/Al_2O_3, Cu/Al_2O_3, and Pd/Al_2O_3 catalysts.

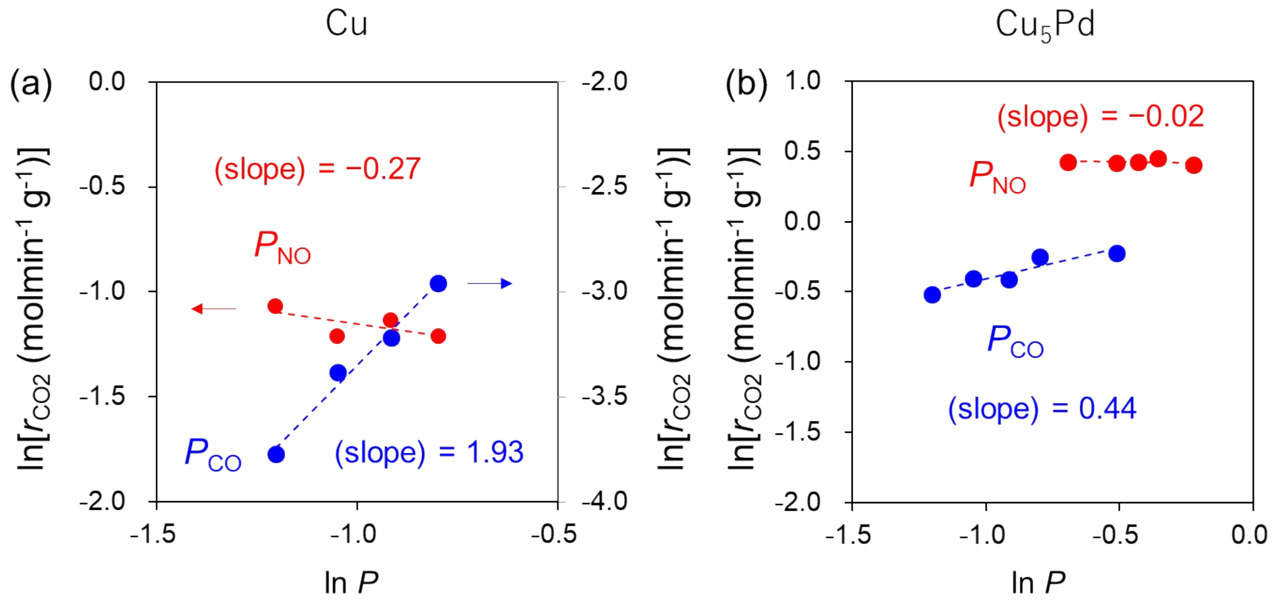

Figure S8 Dependence of reaction rate on NO and CO partial pressures (P_{NO} and P_{CO}, respectively) in NO reduction by CO over Cu/Al_2O_3 and Cu_5Pd/Al_2O_3.

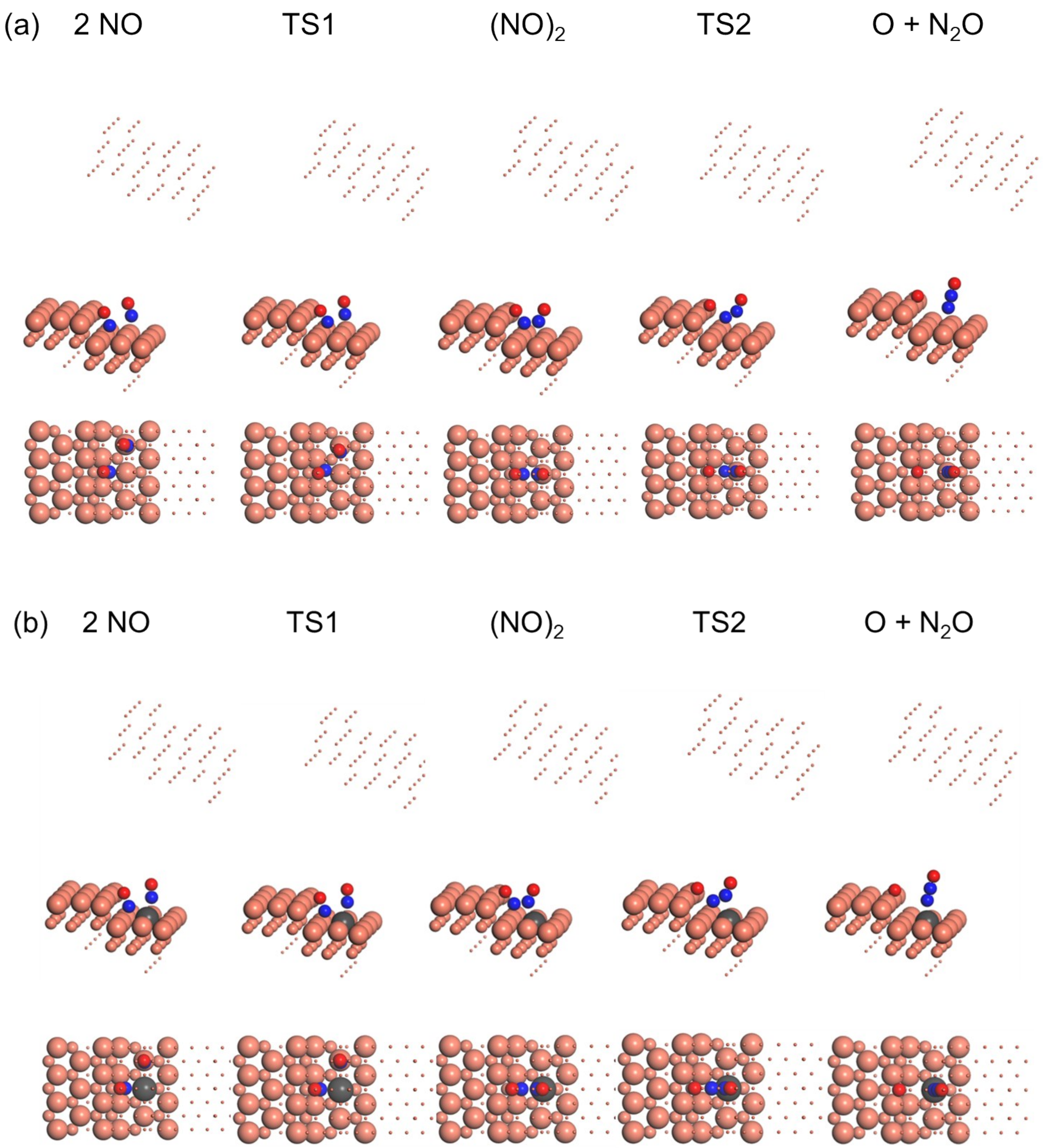

Figure S9. Optimized structures of adsorbates and the corresponding transition states during $(NO)_2$ dimer formation and its subsequent decomposition to N_2O and O over (a) Cu(211) and (b) Pd - substituted Cu(211). For clarity, metal atoms in the sub-surface region are shown as small dots.

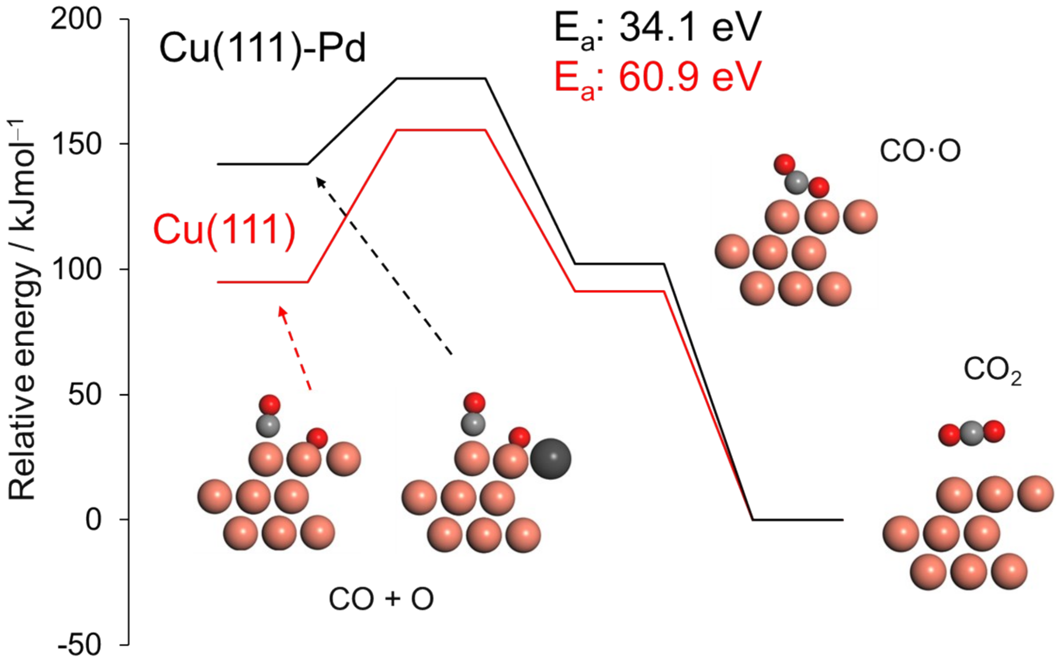

Figure S10. Energy diagrams of CO oxidation over Cu(111) and Pd-substituted Cu(111) surfaces. Total energy of the slab plus free CO_2 was set zero. TS search was performed with the intermediate structure (CO· O) as a tentative final state.

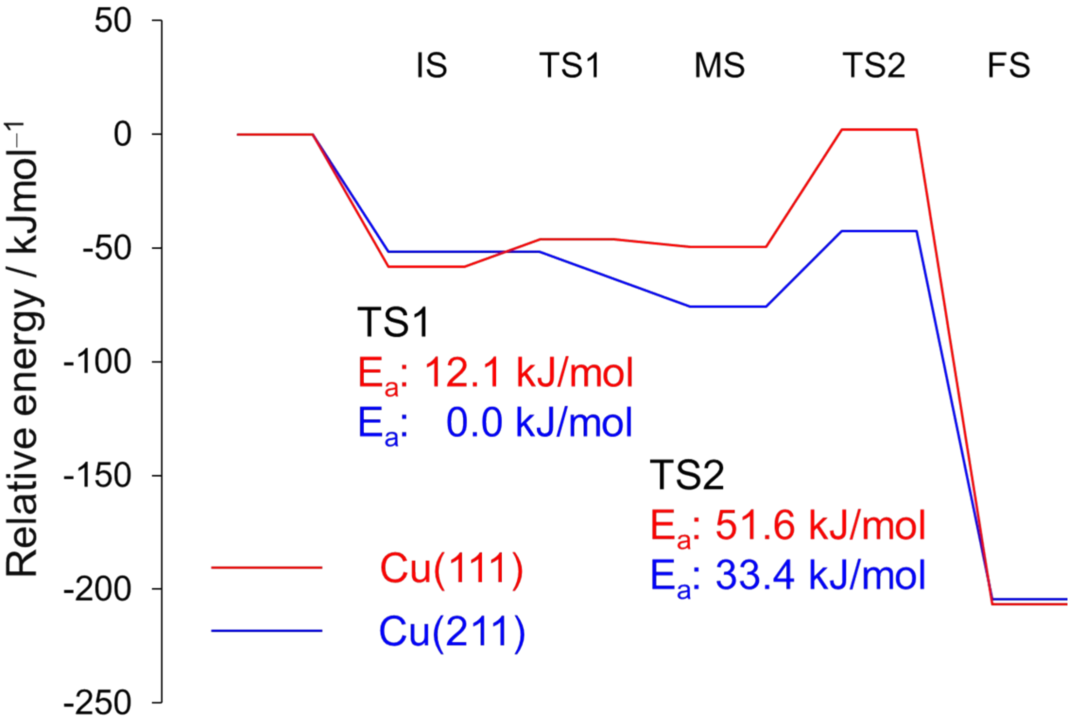

Figure S11. Energy diagrams of N_2O bending (IS→MS) and its subsequent decomposition to N_2 and O (MS→FS) over (a) Cu(111) and (b) Cu(211) surfaces. For Cu(211), conversion from IS to MS was barieer-less. Total energy of slab and free N_2O was set to zero.

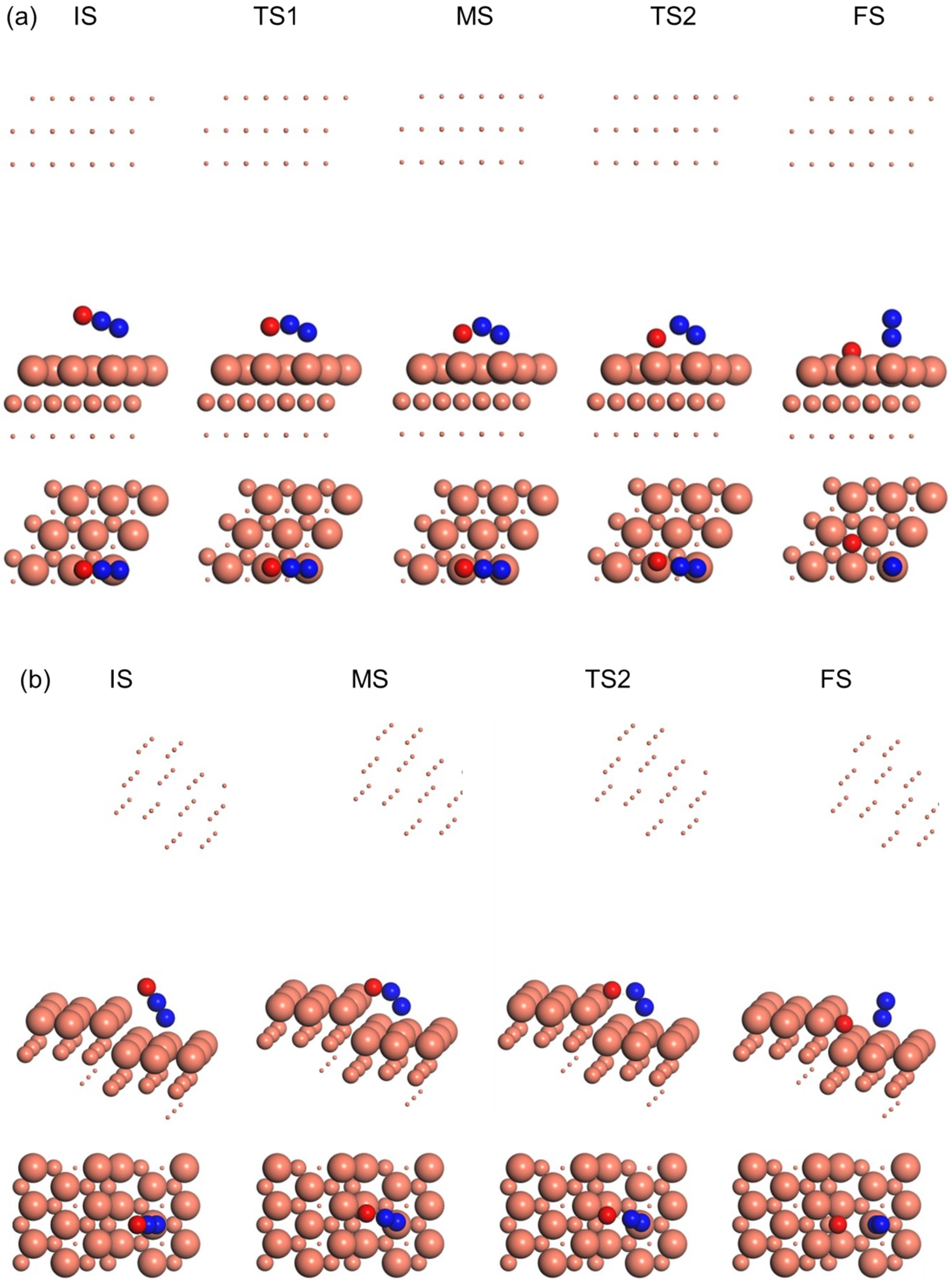

Figure S12. Optimized structures of adsorbates (IS, MS, and FS) and the corresponding transition states (TS1 and TS2) during N_2O bending and its subsequent decomposition to N_2 and O over (a) Cu(111) and (b) Cu(211) surfaces. For clarity, metal atoms in the sub-surface region are shown as small dots.

Kinetic Analysis

A Langmuir-Hinshelwood type mechanism

with an $(NO)_2$ dimer formation and decomposition was considered for NO–CO reaction over Cu-based catalysts as follows:

$NO + \sigma \rightleftarrows NO \cdot \sigma$ (1): NO adsorption

$CO + \sigma \rightleftarrows CO \cdot \sigma$ (2): CO adsorption

$2NO \cdot \sigma \rightleftarrows (NO \cdot \sigma)_2$ (3): NO dimerization

$(NO \cdot \sigma)_2 \rightleftarrows N_2O \cdot \sigma + O \cdot \sigma$ (4): N–O scission

$N_2O \cdot \sigma \rightleftarrows N_2 + O \cdot \sigma$ (5): N_2O decomposition

$CO \cdot \sigma + O \cdot \sigma \rightleftarrows CO_2 + 2\sigma$ (6): CO oxidation

$N_2O \cdot \sigma \rightleftarrows N_2O + \sigma$ (7): N_2O desorption

where, σ indicates an adsorption site. Each step can be regarded to be in equilibrium except when it is the rate-determining step. Therefore, the equilibrium constants are generally defined as follows:

$$K_1 = \theta_{NO}/P_{NO}(1 - \theta)$$

$$K_2 = \theta_{CO}/P_{CO}(1 - \theta)$$

$$K_3 = \theta_{(NO)_2}/{\theta_{NO}}^2$$

$$K_4 = \theta_{N_2O}\theta_O/\theta_{(NO)_2}$$

$$K_5 = P_{N_2}\theta_O/\theta_{N_2O}$$

$$K_6 = P_{CO_2}(1 - \theta)^2/\theta_{CO}\theta_O$$

$$K_7 = P_{N_2O}(1 - \theta)/\theta_{N_2O}$$

where, P_X, θ_X, and $1 - \theta$ are the partial pressure of *X*, coverage of *X*, and percentage of vacant site: $1 - (\theta_{NO} + \theta_{CO} + \theta_{(NO)_2} + \theta_{N_2O} + \theta_O)$, respectively.

Assuming that NO adsorption (1) is the rate determining step, the overall reaction rate can be expressed using a rate constant k as follows:

$$r = kP_{NO}(1 - \theta)$$

here, $1 - \theta$ is expressed using the equilibrium constants and P_X as follows:

$$1 - \theta = \frac{-b \pm \sqrt{b^2 - 4ac}}{2a}$$

where, a, b, and c are expressed as follows: $a = K_3^{-0.5}K_4^{-1.5}K_5^{1.5}K_7^{-2}P_{N_2}^{-1.5}P_{N_2O}^{2}$, $c = -1$

$$b = K_7^{-1}P_{N_2O}\left(K_3^{-0.5}K_4^{-0.5}K_5^{0.5}P_{N_2}^{-0.5} + K_5^{-1}K_6^{-1}K_7^{2}P_{CO_2}P_{N_2}P_{N_2O}^{-2} + K_3P_{N_2}^{-1} + 1\right) + 1$$

Based on these, the reaction orders for P_{NO} (x) and P_{CO} (y) on the overall reaction rate should be described as follows: $x = 1$, $y = 0$.

This indicates the first- and zero-order dependences of r on P_{NO} and P_{CO}, respectively. This does not agree with the experimental results.

Assuming that CO adsorption (2) is the rate determining step, the overall reaction rate can be expressed using a rate constant k as follows:

$$r = kP_{CO}(1 - \theta)$$

here, $1 - \theta$ is expressed using the equilibrium constants and P_X as follows:

$$1 - \theta = \frac{-b \pm \sqrt{b^2 - 4ac}}{2a}$$

where, a, b, and c are expressed those shown in the solution for (1).

Based on these, the reaction orders for P_{NO} (x) and P_{CO} (y) on the overall reaction rate should be described as follows: $x = 0$, $y = 1$.

This indicates the zero- and first-order dependences of r on P_{NO} and P_{CO}, respectively, which is inconsistent with the experimental results.

Next, we assume that NO dimerization (3) is the rate determining step: the overall reaction rate can be expressed using a rate constant k as follows:

$$r = kK_1 {P_{NO}}^2 (1 - \theta)^2$$

here, $1 - \theta$ is expressed using the equilibrium constants and P_X as follows:

$$1 - \theta = \frac{-b \pm \sqrt{b^2 - 4ac}}{2a}$$

where, a, b, and c are expressed as follows: $a = {K_4}^{-1} K_5 {K_7}^{-2} {P_{N_2}}^{-1} {P_{N_2O}}^2$

$b = K_1 P_{NO} + K_2 P_{CO} + {K_7}^{-1} P_{N_2O} \left(K_5 {P_{N_2}}^{-1} + 1 \right) + 1$ $\qquad c = -1$

Based on these, the ranges of the reaction orders for P_{NO} (x) and P_{CO} (y) on the overall reaction rate should be as follows: $2 < x < 4$, $0 < y < 2$. This does not agree with the experimental results with negative x.

Assuming that N–O bond scission of $(NO)_2$ dimer (4) is the rate determining step, the overall reaction rate can be expressed using a rate constant k as follows:

$$r = kK_1^2 K_3 {P_{NO}}^2 (1 - \theta)^2$$

here, $1 - \theta$ is expressed using the equilibrium constants and P_X as follows:

$$1 - \theta = \frac{-b \pm \sqrt{b^2 - 4ac}}{2a}$$

where, a, b, and c are expressed as follows: $a = K_1^2 K_3 {P_{NO}}^2$

$b = K_1 P_{NO} + K_2 P_{CO} + {K_7}^{-1} P_{N_2O} \left(K_5 {P_{N_2}}^{-1} + 1 \right) + 1$ $\qquad c = -1$

Based on these, the ranges of the reaction orders for P_{NO} (x) and P_{CO} (y) on the overall reaction rate should be as follows: $-2 < x < 0$, $0 < y < 2$, which is well consistent with the experimental orders for both Cu/Al_2O_3 and Cu_5Pd/Al_2O_3.

Then, we assume that N_2O decomposition (5) is the rate determining step, affording the overall reaction rate expressed as follows:

$$r = kK_7^{-1}P_{N_2O}(1-\theta)$$

here, $1-\theta$ is expressed using the equilibrium constants and P_X as follows:

$$1-\theta = \frac{-b \pm \sqrt{b^2 - 4ac}}{2a}$$

where, a, b, and c are expressed as follows: $a = K_2^{-1}K_4^{-1}K_6^{-1}K_7^{-1}P_{N_2O}P_{CO_2}P_{CO}^{-1}$

$$b = K_1P_{NO} + K_2P_{CO} + K_7^{-1}P_{N_2O}\left(K_5P_{N_2}^{-1} + 1\right) + 1 \qquad c = -1$$

Based on these, the ranges of the reaction orders for P_{NO} (x) and P_{CO} (y) on the overall reaction rate should be as follows: $0 < x < 1$, $0 < y < 2$, which is inconsistent with the experimental results with negative x.

When CO oxidation (6) is assumed as the rate determining step, the overall reaction rate is expressed as follows:

$$r = kK_2K_5K_7^{-1}P_{N_2}^{-1}P_{N_2O}P_{CO}(1-\theta)^2$$

here, $1-\theta$ is expressed using the equilibrium constants and P_X as follows:

$$1-\theta = \frac{-b \pm \sqrt{b^2 - 4ac}}{2a}$$

where, a, b, and c are expressed those shown in the solution for (3).

Based on these, the ranges of the reaction orders for P_{NO} (x) and P_{CO} (y) on the overall reaction rate should be as follows: $0 < x < 2$, $1 < y < 3$. This does not agree with the experimental results.

When N_2O desorption (7) is assumed as the rate determining step, the overall reaction rate is expressed as follows:

$$r = kK_2^{-1}K_5^{-1}K_6^{-1}P_{N_2}P_{CO_2}P_{CO}^{-1}(1 - \theta)$$

here, $1 - \theta$ is expressed using the equilibrium constants and P_X as follows:

$$1 - \theta = \frac{-b \pm \sqrt{b^2 - 4ac}}{2a}$$

where, a, b, and c are expressed as follows: $a = K_2^{-1}K_4^{-1}K_5^{-1}K_6^{-1}P_{N_2}P_{CO_2}^{2}P_{CO}^{-2}$

$$b = K_1P_{NO} + K_2P_{CO} + K_2^{-1}K_6^{-1}P_{CO_2}\left(K_5^{-1}P_{N_2} + 1\right)P_{CO}^{-1} + 1 \qquad c = -1$$

Based on these, the ranges of the reaction orders for P_{NO} (x) and P_{CO} (y) on the overall reaction rate should be as follows: $0 < x < 1$, $1 < y < 3$. This does not agree with the experimental results.

Thus, assuming N–O bond scission of $(NO)_2$ dimer (4) as the rate-determining step exclusively gave the reaction order ranges consistent with the experiment. On the basis of these results, we concluded that the rate-determining step of NO–CO reaction over Cu-based catalysts is N–O bond scission of $(NO)_2$ dimer.

Appendix Table 1. Summary of reaction orders

RDS in NO–CO	$r = kP_{NO}^{x}P_{CO}^{y}$	
(1)	$x = 1$	$y = 0$
(2)	$x = 0$	$y = 1$
(3)	$2 < x < 4$	$0 < y < -2$
(4)	$-2 < x < 0$	$0 < y < 2$

(5)	$0 < x < 1$	$0 < y < 2$
(6)	$0 < x < 2$	$1 < y < 3$
(7)	$0 < x < 1$	$1 < y < 3$
